# Development of prediction models for carbapenem-resistant *Klebsiella pneumoniae* acquisition and prognosis in adult patients

**DOI:** 10.3389/fphar.2024.1439116

**Published:** 2024-11-05

**Authors:** Huijuan Yao, Yu Yang, Huimin Yao, Shuhong Bu, Lixia Li, Fang Wang, Jian Zhang, Jihui Chen

**Affiliations:** ^1^ Department of Pharmacy, Xinhua Hospital, School of Medicine, Shanghai Jiao Tong University, Shanghai, China; ^2^ School of Traditional Chinese Medicine, Jilin Agriculture Science and Technology University, Jilin, China

**Keywords:** carbapenem-resistant, *Klebsiella pneumoniae*, infection, nomogram, prognosis

## Abstract

**Objectives:**

To explore the risk factors and clinical outcomes of carbapenem-resistant *Klebsiella pneumoniae* (CRKP) infection and establish nomograms to predict the probability of CRKP infection and mortality in adult patients.

**Methods:**

Patients infected with KP from August 2019 to April 2021 in a tertiary hospital in Shanghai were enrolled. Risk factors associated with CRKP and 30-day mortality were identified using multivariate logistic regression analysis and Cox regression analysis.

**Results:**

Overall, 467 patients with KP infection were enrolled, wherein 210 (45.0%) patients were infected with CRKP and 257 (55.0%) patients with carbapenem-susceptible *K. pneumoniae* (CSKP). Five factors, namely Charlson’s Comorbidity Index (CCI) ≥ 3, the use of central venous catheterization, prior hospitalization during the 3 months before infection, and previous exposure to carbapenems and broad-spectrum β-lactams, were found to be independently associated with CRKP infection. Based on these parameters, the nomogram showed a better performance as indicated by C-index of 0.94 (95% confidence interval [CI]: 0.92–0.96) and well-fitted calibration curves. CRKP was independently associated with 30-day mortality. Multivariate Cox regression analysis revealed that age ≥65 years, higher CCI scores, higher Sequential Organ Failure Assessment scores, the presence of respiratory failure, albumin levels ≤30 g/L, and non-appropriate treatments in 3 days, were associated with 30-day mortality.

**Conclusion:**

The predictive nomogram established in this study can facilitate the clinicians to make better clinical decisions when treating patients with KP infection.

## Introduction

*Klebsiella pneumoniae* (KP) is emerging as one of the most common Gram-negative pathogens causing a significant amount of mortality and morbidity in hospitals, with a wide variety of infections (Bengoechea and Sa Pessoa, 2019; Rodríguez-Medina et al., 2019). In clinical practice, carbapenems, broad-spectrum β-lactams, fluoroquinolones and aminoglycosides are commonly used antibiotics to treat severe KP infections. However, with the increasing prevalence of carbapenem-resistant *K. pneumoniae* (CRKP), treatment options have become severely limited, leading to the use of alternative agents like tigecycline, polymyxin B, and ceftazidime-avibactam. During the last 10-year period in China, the cases of CRKP have increased from 14.1% in 2013 to 26.0% in 2023 (http://www.chinets.com/Data/GermYear). Due to high mortality rates and the limited antibiotic therapy options, CRKP infections have emerged as an important problem to public health (Durante-Mangoni et al., 2019; Tilahun et al., 2021). Recent studies demonstrated the association of CRKP with prolonged hospital stays, increased medical costs, and higher mortality for patients (Del Puente et al., 2019).

Since CRKP infection can lead to fatal clinical outcomes, early and accurate evaluation is essential in managing its treatment. Several studies so far have evaluated the risk factors contributing to the acquisition of CRKP and mortality (Li et al., 2020; Xiao et al., 2020; Wu et al., 2022) by simply analyzing the associated factors; however, further predictive models are warranted to identify high-risk patients for clinical applications. Moreover, metagenomic next-generation sequencing (mNGS), a powerful method for pathogen detection, is increasingly being used. This method showed higher sensitivity and specificity, which could markedly improve the rate of early diagnosis of KP infection (Forbes et al., 2017; Leguia et al., 2020). However, mNGS cannot determine the antibiotic resistance of causative pathogens accurately relative to that by the conventional culture methods (Liu et al., 2022), which emphasizes the need for personalized prediction models.

Recently, the nomogram has been widely used to visualize the predictive model (Park, 2018). In this retrospective study, we conducted an extensive analysis of the clinical data pertaining to adult patients afflicted with KP infection. Utilizing this data, we successfully formulated a practical and operable nomogram scoring system. This system is designed to assist clinicians in accurately predicting the likelihood of CRKP infection, as well as the associated prognosis, thereby contributing to more informed and targeted therapeutic interventions.

## Materials and methods

### Study design

The present retrospective study was carried out in a 2600-bed tertiary-care hospital, conducted from August 2019 to October 2021. Data from August 2019 to March 2021 were used for modeling, while data from April 2021 to October 2021 were used for validation. Patients who were 18 years of age and older, with proven KP infection (defined as the presence of a positive culture, associated with clinical signs of systemic inflammatory response syndrome) were enrolled. Individuals exhibiting KP colonization (characterized by positive KP cultures, yet devoid of any clinical or laboratory evidence of infection) or those engaged in an end-of-life care pathway were excluded from the study.

### Definitions

The relevant demographic and clinical data on the day of KP detection were extracted and reviewed from patients’ charts and electronic medical records and documented in the data collection form.

Comorbid conditions were quantified using the Charlson’s Comorbidity Index (CCI), a widely used tool that predicts mortality risk by assigning weighted scores to comorbidities, with higher scores indicating worse outcomes (Charlson et al., 1994). The use of immunosuppressant was defined as receipt of corticosteroids at a dosage equivalent to or higher than 10 mg of prednisolone daily for more than 2 weeks, or antineoplastic chemotherapy within 4 weeks prior to the onset of KP infection. Respiratory failure was defined as a partial pressure of arterial oxygen/fraction of inspired oxygen PaO_2_/FiO_2_ ratio <200 mmHg, and/or the need for mechanical ventilation (either non-invasive positive pressure ventilation or invasive mechanical ventilation). Antimicrobial drug exposure was defined as the use of antibiotics for >72 h within the 30-day prior to KP infection. Appropriate therapy in 3 days was defined as treatment that was initiated before or within 72 h, with at least one antimicrobial agent that has been proven *in vitro* activity against the KP strains.

### Antimicrobial susceptibility testing

KP isolates were identified using matrix-assisted laser desorption ionization-time of flight mass spectrometry (MALDI-TOF), and antimicrobial minimum inhibitory concentrations (MICs) were determined using the Vitek 2 system (bioMerieux, France) and the AST-GN card, as described previously (Chen et al., 2021). Carbapenem resistance was defined as resistant to any carbapenem antimicrobial (a MIC of ≥2 μg/mL for ertapenem or a MIC of ≥4 μg/mL for imipenem/meropenem) in accordance with the CLSI criteria (CALS Institute, 2021).

### Statistical analysis

Statistical analyses were performed with R version 4.1.0 (R Foundation for Statistical Computing, Vienna, Austria). More than 10% of the missing variables were excluded from the analyses. The remaining missing values were interpolated via multiple interpolations (MICE package). Continuous variables were presented as mean ± standard deviation or medians (interquartile range [IQR]), as appropriate, and categorical variables were reported as numbers and percentages. All categorical variables were compared by using the Fisher’s exact test or Chi-square test, and continuous variables were compared using the Student’s t-test or the Mann-Whitney U test, if appropriate. All statistical tests were performed two-tailed, and a *P*-value <0.05 was considered to indicate statistical significance.

Univariate and multivariate analyses were performed to identify independent risk factors, and the LASSO regression was applied to reduce the data dimensions (glmnet package). Odds ratio (OR) or hazard ratio (HR) with a 95% confidence interval (CI) was employed for measuring the strength of the association. The nomogram for predicting the probability of CRKP infection and prognosis were established based on the logistic regression and Cox regression analyses (rms package), respectively. The predictive performance was evaluated in terms of its discriminative ability, calibration, and clinical usefulness (Steyerberg and Vergouwe, 2014). The bootstrap method was used for repeated sampling 1,000 times to internally evaluate the consistency of model prediction, and the discriminative ability and predictive accuracy of the model were assessed using the area under (AUC) the receiver operating characteristic curve (ROC) and Harrel’s concordance index (C-index), using the “timeROC” and “pROC” R packages. A decision curve analysis (DCA) was employed to evaluate the clinical usefulness and net benefit of the intervention, utilizing the ggDCA package. An online version of the nomogram was established and deployed with reference to the shinyapps online website using “DynNom” and “rsconnect” R packages.

Propensity score matching (PSM) analysis was applied to balance the baseline characteristics between the CRKP and CSKP groups in the ratio of 1:2 (MatchIt package), in accordance with the differences in sex, age, baseline diseases or comorbidities, CCI, clinical status, invasive procedure, and the type of infections. The 30-day survival rate was generated using Kaplan–Meier (K-M) curve and the differences obtained were compared using the log-rank test.

## Results

### Characteristics of patients with KP

A total of 467 patients with KP infection were enrolled in this study, wherein 210 patients were infected with carbapenem-resistant KP (CRKP) while 257 were with carbapenem-susceptible KP (CSKP), and no significant changes were observed in the proportions of CRKP from 2019 to 2021. The demographic details, clinical characteristics, and laboratory findings were provided in Table 1. The median age was 67 (IQR, 59–77) years and 68.7% of the patients were of the male gender. The detailed antimicrobial susceptibility results are presented in Table 2. 86 patients were included in the validation group, with relevant data available in Supplementary Table S1.

**TABLE 1:** Characteristics of the study patients with *Klebsiella pneumoniae* infection.

Characteristic	All patients n = 467	CRKP infection n = 210 (45.0%)	CSKP infection n = 257 (55.0%)	*P* value
Patient variables				
Male sex, n (%)	321 (68.7)	154 (73.3)	167 (65)	0.053
Age, y, median (IQR)	67 (59, 77)	71 (61, 81)	66 (56, 73)	<0.001
Baseline disease or comorbidity, n (%)				
Diabetes mellitus	153 (32.8)	63 (30.0)	90 (35.0)	0.251
Cardiovascular disease	208 (44.5)	133 (63.3)	75 (29.2)	<0.001
Cerebrovascular disease	172 (36.8)	107 (51.0)	65 (25.3)	<0.001
Renal disease	86 (18.4)	55 (26.2)	31 (12.1)	<0.001
Hematological disease	23 (4.9)	15 (7.1)	8 (3.1)	0.051
Digestive diseases	153 (32.8)	64 (30.5)	89 (34.6)	0.342
Malignant solid tumor	110 (23.6)	39 (18.6)	71 (27.6)	0.023
Prior surgery^a^	268 (57.4)	143 (68.1)	125 (48.6)	<0.001
Immunosuppressant use	63 (13.5)	24 (11.4)	39 (15.2)	0.240
ICU admission	276 (59.1)	174 (82.9)	102 (39.7)	<0.001
CCI, median (IQR)	3 (2, 5)	4 (3, 6)	2 (1, 3)	<0.001
CCI≥3, n (%)	275 (58.9)	174 (82.9)	101 (39.3)	<0.001
Clinical status, n (%)				
Respiratory failure	70 (15.0)	51 (24.3)	19 (7.4)	<0.001
Heart failure	87 (18.6)	51 (24.3)	36 (14)	0.005
MODS	28 (6.0)	19 (9.0)	9 (3.5)	0.015
SOFA score, median (IQR)	3 (0, 5)	3 (3, 5.75)	1 (0, 4)	<0.001
Invasive procedure, n (%)				
Mechanical ventilation	229 (49.0)	157 (74.8)	72 (28.0)	<0.001
Central venous catheterization	285 (61.0)	194 (92.4)	91 (35.4)	<0.001
Urinary catheterization	325 (69.6)	183 (87.1)	142 (55.3)	<0.001
Gastric catheterization	259 (55.5)	175 (83.3)	84 (32.7)	<0.001
CRRT	27 (5.8)	20 (9.5)	7 (2.7)	0.003
Type of infections, n (%)				
Pneumonia	262 (56.1)	131 (62.4)	131 (51.0)	0.014
Intra-abdominal infection	32 (6.9)	18 (8.6)	14 (5.4)	0.187
Urinary tract infection	89 (19.1)	31 (14.8)	58 (22.6)	0.034
Gastrointestinal infection	37 (7.9)	3 (1.4)	34 (13.2)	<0.001
Primary bloodstream infection	28 (6.0)	18 (8.6)	10 (3.9)	0.039
Skin and soft-tissue infection	15 (3.2)	5 (2.4)	10 (3.9)	0.362
Catheter-related infection	9 (1.9)	7 (3.3)	2 (0.8)	0.085
CNS infection	2 (0.4)	1 (0.5)	1 (0.4)	1
Laboratory variables from blood, Mean ± SD				
WBC, × 10^9^/L	11.16 ± 5.45	11.2 ± 5.73	11.13 ± 5.23	0.888
ANC, × 10^9^/L	11.07 ± 12.72	13.33 ± 17.86	9.23 ± 5.16	0.003
Lymphocyte, × 10^9^/L	4.92 ± 6.28	9.55 ± 6.94	1.13 ± 0.72	<0.001
Hemoglobin, g/L	104 ± 23	96 ± 21	111 ± 22	<0.001
Platelet, × 10^9^/L	218 ± 123	216 ± 131	219 ± 116	0.765
C-reactive protein, mg/L	82 ± 62	83 ± 60	81 ± 64	0.773
Albumin, g/L	33.3 ± 5.7	32.5 ± 5.5	33.89 ± 5.9	0.009
Hospital stay before KP infection, days, median (IQR)	9 (4, 17.5)	13.5 (7.25, 22.75)	6 (4, 11)	<0.001
Previous exposure to antibiotics^a^, n (%)				
Carbapenems	129 (27.6)	95 (45.2)	34 (13.2)	<0.001
Broad-spectrum β-lactams^b^	209 (44.8)	133 (63.3)	76 (29.6)	<0.001
First and second-generation cephalosporins	14 (3)	1 (0.5)	13 (5.1)	0.974
Fluoroquinolones	62 (13.3)	28 (13.3)	34 (13.2)	0.011
Aminoglycosides	12 (2.6)	11 (5.2)	1 (0.4)	0.002
Tigecyclines	20 (4.3)	15 (7.1)	5 (1.9)	0.976
Glycopeptides	20 (4.3)	0 (0)	20 (7.8)	<0.001
Fosfomycins	12 (2.6)	3 (1.4)	9 (3.5)	0.240
Tetracyclines	8 (1.7)	7 (3.3)	1 (0.4)	0.025
Polymyxin B	4 (0.9)	3 (1.4)	1 (0.4)	0.331
Others^c^	11 (2.3)	0 (0)	11 (4.3)	0.005
Prior hospitalization^d^, n (%)	207 (44.3)	139 (66.2)	68 (26.5)	<0.001
30-day mortality, n (%)	84 (18)	68 (32.4)	16 (6.2)	<0.001
Total hospitalisation cost^e^, USD, Mean ± SD	26,440 ± 38,645	38,786 ± 49,151	16,353 ± 22,783	<0.001

Notes:

^a^ During the 30 days before *Klebsiella pneumoniae* infection.

^b^ Broad-spectrum β-lactams including third- and fourth-generation cephalosporins, beta-lactam: beta-lactamase inhibitor combinations.

^c^ Other drugs include sulfamethoxazole-trimethoprim, aztreonam, macrolides, clindamycin, and penicillin.

^d^ During the 3 months before *Klebsiella pneumoniae* infection.

^e^ USD1 = CNY6.5 in year 2021.

Abbreviations: CRKP, carbapenem-resistant *Klebsiella pneumoniae*; CRKP, carbapenem-susceptible *Klebsiella pneumoniae*; IQR, interquartile Range; MODS, multiple organ dysfunction syndrome; ICU, intensive care unit; CCI, charlson comorbidity index; SOFA, sequential organ failure assessment; CNS, central nervous system; CRRT, continuous renal replacement therapy; WBC, white blood count; ANC, absolute neutrophil count.

**TABLE 2:** Antimicrobial susceptibility distributions of CRKP and CSKP isolates.

Antibiotics	CRKP (n = 210)			CSKP (n = 257)		
	S	I	R	S	I	R
AK	103 (48.9%)	0	107 (51.1%)	250 (97.3%)	7 (2.7%)	0
CAZ	0	0	210 (100%)	214 (83.5%)	6 (2.3%)	37 (14.2%)
CMZ	2 (0.7%)	11 (5.5%)	197 (93.8%)	238 (92.6%)	1 (0.4%)	18 (7.0%)
SCF	0	2 (0.7%)	208 (99.3%)	207 (80.4%)	38 (14.6%)	13 (5.0%)
CTX	0	0	210 (100%)	177 (68.8%)	5 (1.9%)	75 (29.2%)
ROX	0	0	210 (100%)	168 (65.5%)	6 (2.3%)	83 (32.2%)
GEN	51 (24.5%)	1 (0.4%)	158 (75.2%)	220 (85.8%)	0	37 (14.2%)
LEV	5 (2.6%)	2 (1.1%)	202 (96.4%)	162 (62.9%)	59 (22.8%)	37 (14.3%)
CIP	5 (2.2%)	0	205 (97.8%)	169 (65.6%)	10 (3.9%)	78 (30.5%)
CRO	1 (0.4%)	0	209 (99.6%)	186 (72.4%)	0	71 (27.6%)
FEP	0	0	210 (100%)	230 (89.6%)	0	27 (10.4%)
IPM	0	0	210 (100%)	256 (99.6%)	1 (0.4%)	0
MEM	0	0	210 (100%)	257 (100%)	0	0
TGC	205 (97.4%)	2 (1.1%)	3 (1.5%)	257 (100%)	0	0
PB	82 (39.1%)	119 (56.5%)	9 (4.3%)	257 (100%)	0	0
FOT	100 (47.8%)	25 (11.7%)	85 (40.5)	257 (100%)	0	0
SXT	79 (37.6%)	0	131 (62.4%)	196 (76.2%)	0	61 (23.8%)
CZA	184 (87.5%)	0	26 (12.5%)	257 (100%)	0	0
TZP	1 (0.4%)	1 (0.4%)	208 (99.3%)	240 (93.5%)	13 (5.0%)	4 (1.5%)
SAM	0	0	210 (100%)	162 (63.1%)	11 (4.2%)	84 (32.7%)

Abbreviations: amikacin (AK), ceftazidime (CAZ), cefmetazole (CMZ), cefoperazone-sulbactam (SCF), cefotaxime (CTX), cefuroxime (ROX), gentamicin (GEN), levofloxacin (LEV), ciprofloxacin (CIP), ceftriaxone (CRO), cefepime (FEP), imipenem (IPM), meropenem (MEM), tigecycline (TGC), polymyxin B (PB), fosfomycin (FOT), sulfamethoxazole-trimethoprim (SXT), ceftazidime avibatan (CZA), piperacillin-tazobactam (TZP), ampicillin-sulbactam (SAM).

### Risk factors associated with CRKP infection and prediction model

The CRKP group had higher values for age, CCI, and Sequential Organ Failure Assessment (SOFA) score, whereas lower values for hemoglobin, albumin, and platelet counts. CRKP was more likely to be observed in patients with ICU admission, longer hospital stays, surgery within the last 30 days, and prior hospitalization during the 3-month before KP infection. With reference to the treatment measurements, the patients in the CRKP group showed greater use of mechanical ventilation, central venous catheterization, urinary catheterization, gastric catheterization, and continuous renal replacement therapy. Moreover, the patients in the CRKP group recorded a higher frequency of previous antibiotic exposure, including those of carbapenems, broad-spectrum β-lactams, fluoroquinolones, aminoglycosides, glycopeptides, and tetracyclines.

To eliminate the collinearity of the variables and avoid over-fitting, the LASSO regression was employed to reduce the data dimensionality. When the number of variables was reduced to 6, the model conferred good performance, and it was deemed easy to implement. Multivariate logistic regression analysis identified the followings as independent risk factors for CRKP infection: CCI ≥3 (OR, 7.77; 95% CI: 4.18–15.00; *P* < 0.001), the use of central venous catheterization (OR, 25.87; 95% CI: 12.69–57.13; *P* < 0.001), prior hospitalization during the last 3 months before infection (OR, 5.01; 95% CI: 2.68–9.67; *P* < 0.001), and previous exposure to carbapenems (OR, 5.29; 95% CI: 2.78–10.43; *P* < 0.001) and broad-spectrum β-lactams (OR, 7.34; 95% CI: 3.80–14.76; *P* < 0.001) (Table 3).

**TABLE 3:** Final multivariable analysis for predicting the risk of CRKP infection.

Variable	OR	95% CI	*P* value
CCI, ≥3 vs. < 3	7.77	4.18–15.00	<0.001
Central venous catheterization	25.87	12.69–57.13	<0.001
Prior hospitalization^a^	5.01	2.68–9.67	<0.001
Previous exposure to antibiotics^b^			
Carbapenems	5.29	2.78–10.43	<0.001
Broad-spectrum β-lactams^c^	7.34	3.80–14.76	<0.001

Notes:

^a^ During the 3 months before *Klebsiella pneumoniae* infection.

^b^ During the 30 days before *Klebsiella pneumoniae* infection.

^c^ Broad-spectrum β-lactams including the third- and fourth-generation cephalosporins, beta-lactam: beta-lactamase inhibitor combinations.

Abbreviations: CRKP, carbapenem-resistant *Klebsiella pneumoniae*; OR, odds ratio; CI, confidence interval; CCI, charlson comorbidity index.

**FIGURE 1:**
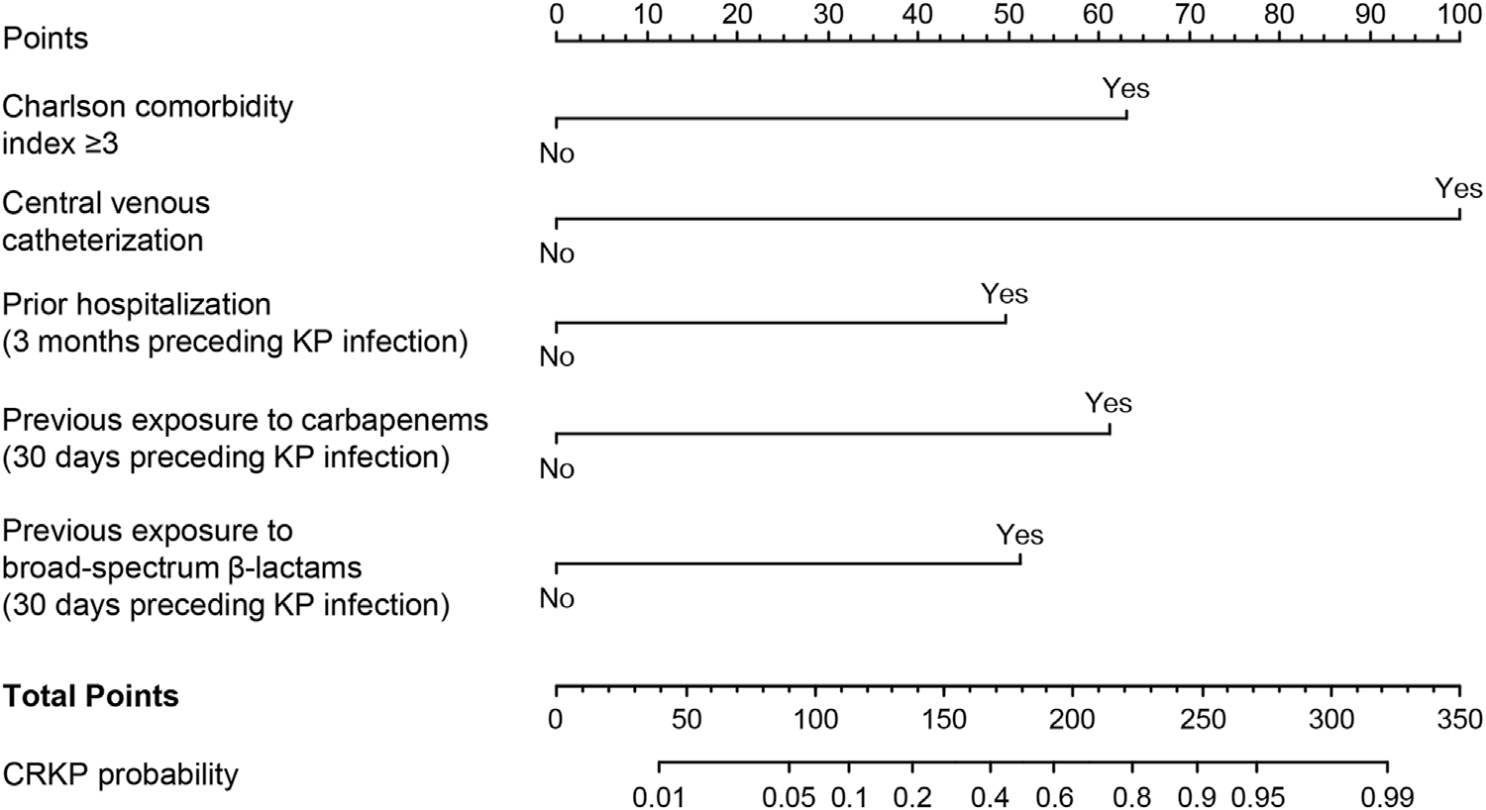
Nomogram for predicting the probability of carbapenem-resistant *Klebsiella pneumoniae* in adult patients. Points are summed for each risk factor. Broad-spectrum β-lactams included third- and fourth-generation cephalosporins, beta-lactam: beta-lactamase inhibitor combinations.

**FIGURE 2:**
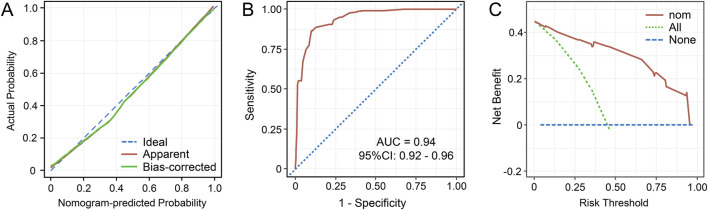
Calibration curves for the nomogram to predict the probability of carbapenem-resistant *Klebsiella pneumoniae* in adult patients **(A)**. The receiver operating characteristics (ROC) curves **(B)** and decision curve analysis (DCA) **(C)** of the prediction model. Abbreviations: AUC, the area under the curve; CI, confidence interval; nom, nomogram.

The abovementioned independent factors were employed to establish a nomogram to predict the probability of CRKP infection (Figure 1). The C-index and Brier score of the model indicated its good accuracy (C-index 0.94, 95% CI 0.92–0.96; Brier score 0.093). The calibration plots and ROC curves were utilized to validate the predictive accuracy of the model (Figures 2A, B). The nomogram showed a superior net benefit for predicting CRKP in the DCA (Figure 2C). Furthermore, 86 patients were used for external validation of the model. The C-index for the validation set was 0.915 (95% CI 0.857–0.973), and the Brier score was 0.131. The calibration plots indicated that the external validation model was well-calibrated (Supplementary Figure S1). Finally, we developed a dynamic nomogram which is accessible for free on https://jihuichen.shinyapps.io/dynnomapp_for_probability/ (Supplementary Figure S2).

### CRKP infection was independently associated with mortality

**FIGURE 3:**
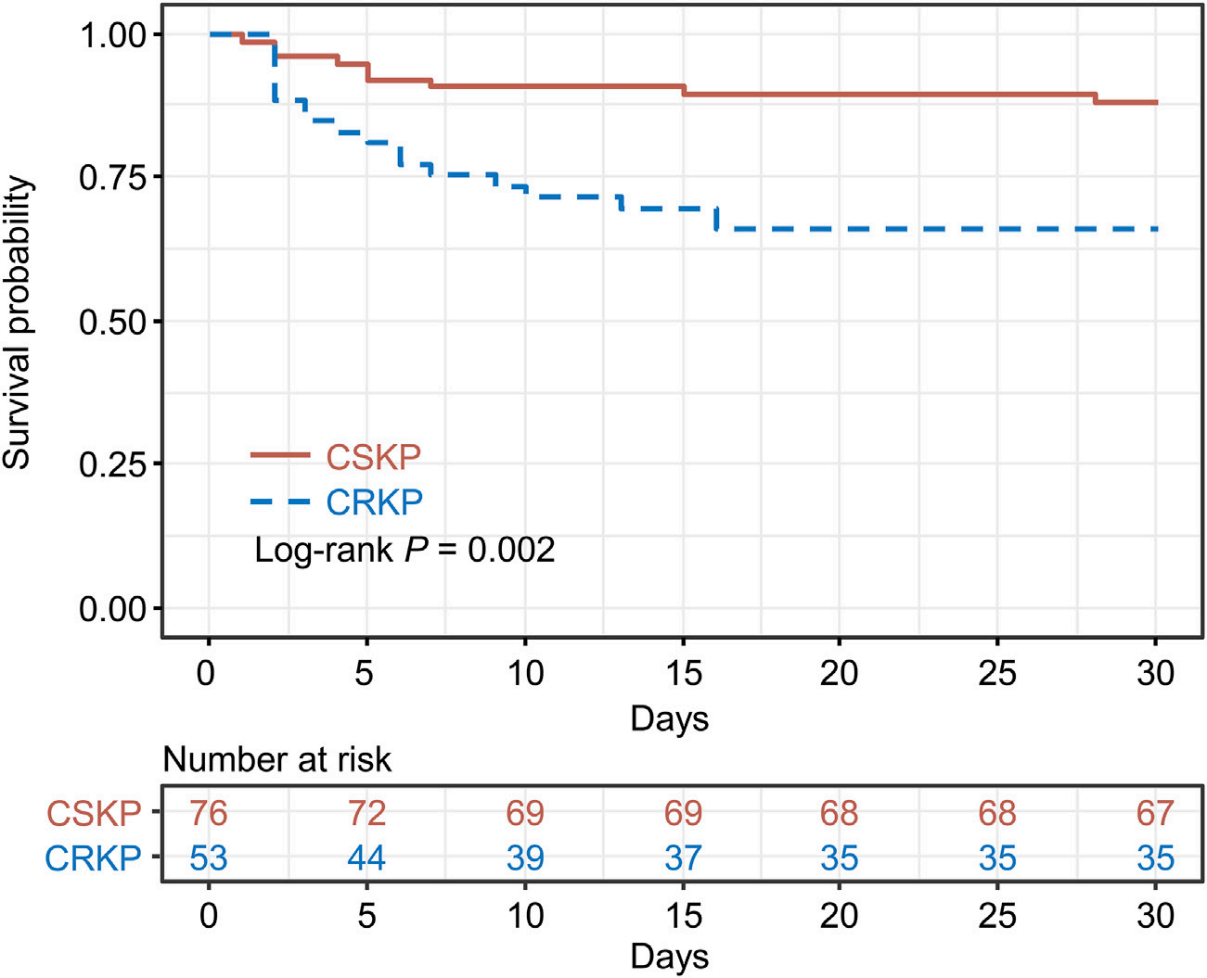
Kaplan–Meier’s survival estimation of the 30-day survival probability of carbapenem-resistant *Klebsiella pneumoniae* (CRKP) and carbapenem-susceptible *Klebsiella pneumoniae* (CSKP) patients. Propensity score matching was conducted between the CRKP and CSKP groups, and the CRKP and CSKP groups were matched on propensity scores.

PSM analysis was conducted between the CRKP and CSKP groups to estimate the impact of CRKP infection on the 30-day survival rate. After PSM, the 2 groups indicated no significant difference (*P* > 0.05) in terms of all baseline characteristics and were comparable (Supplementary Table S2). However, the K-M survival analysis indicated significant differences between the CRKP and CSKP groups in terms of the 30-day survival rate (*P* = 0.002) (Figure 3).

### The mortality risk prediction model and nomogram

The demographic details, clinical characteristics, and laboratory findings were compared between the 30-day survivors and non-survivors (Supplementary Table S3). The 30-day mortality rates were found to be 18% (84/467) in the entire study population, 32.4% (68/210) among CRKP patients, and 6.2% (16/257) among CSKP patients.

**FIGURE 4:**
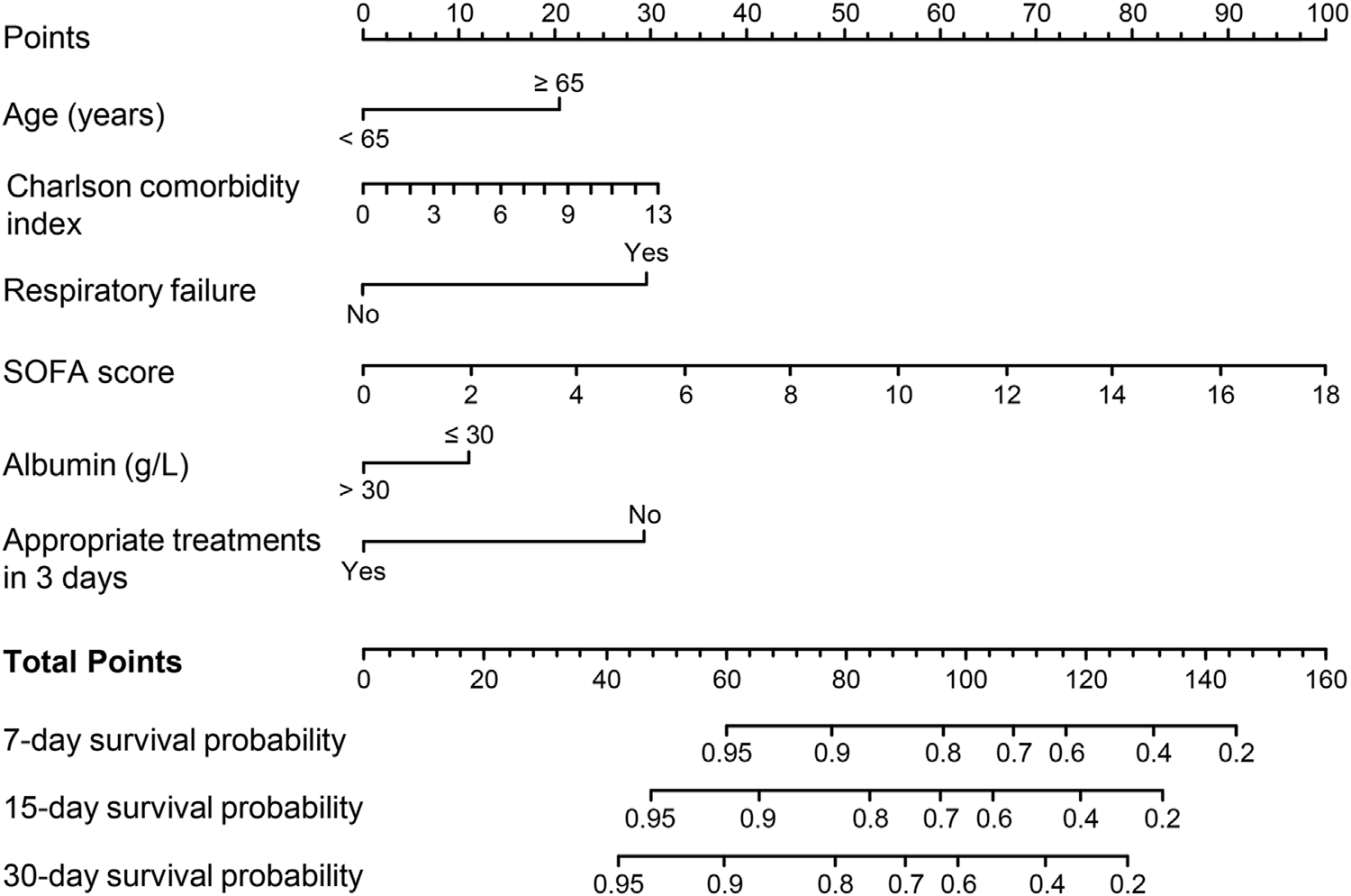
Nomogram for predicting 7-, 15-, and 30-day mortality in adult patients with *Klebsiella pneumoniae* infection. Abbreviations: SOFA, Sequential Organ Failure Assessment.

**FIGURE 5:**
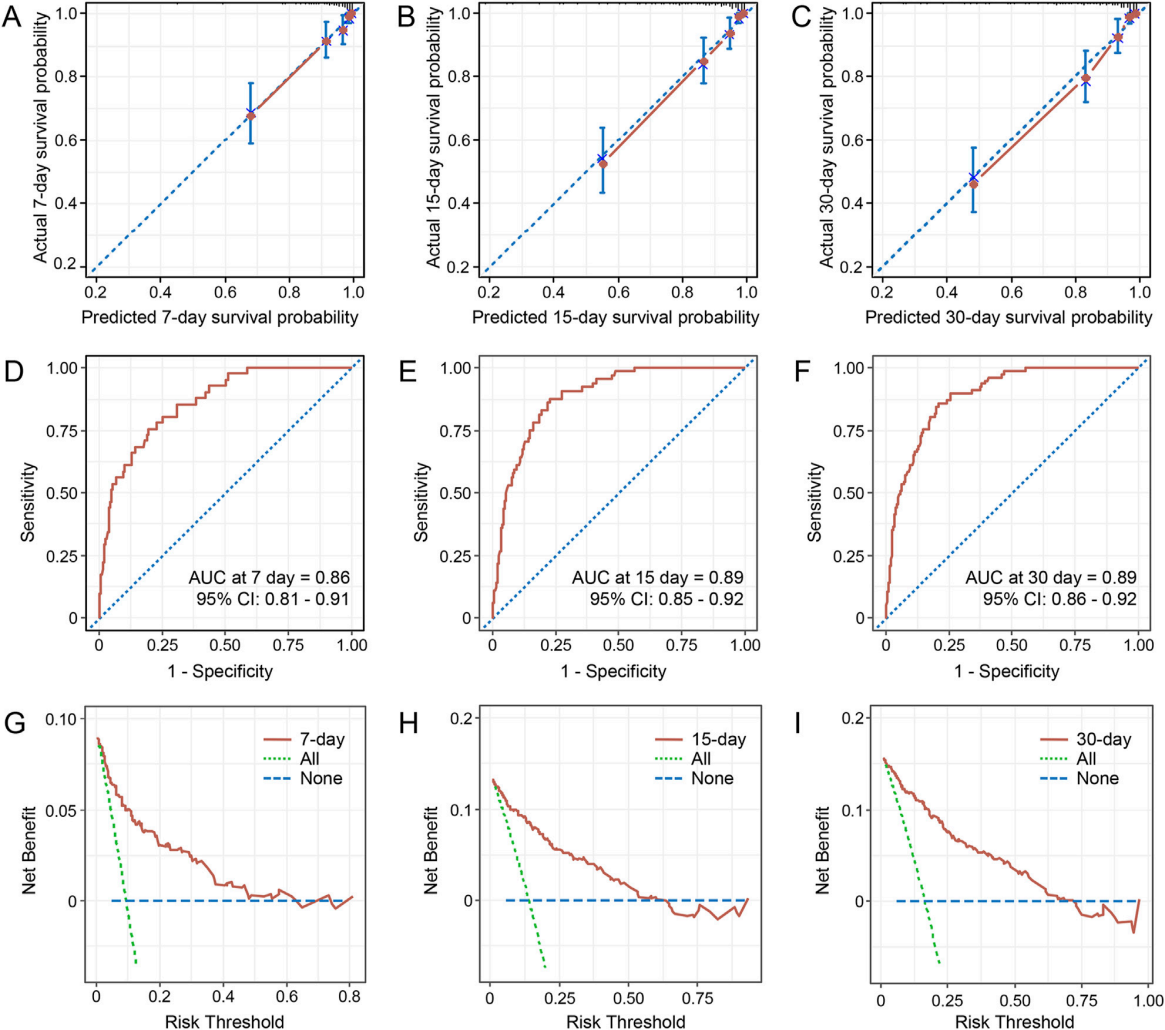
Calibration curves for the nomogram predicting 7-, 15-, and 30-day survival probability **(A–C)**. The time-dependent receiver operating characteristics (ROC) curves for predicting 7-, 15-, and 30-day survival **(D–F)**. Decision curve analysis for the 7-, 15-, and 30-day survival nomogram **(G–I)**. Abbreviations: AUC, the area under the curve; CI, confidence interval.

Variables with P < 0.05 in univariate Cox regression analyses are shown in Table 4. The LASSO regression was applied, and six variables were selected into the final model. Multivariate Cox regression analysis revealed age ≥65 years, higher CCI, presence of respiratory failure, higher SOFA score, albumin ≤30 g/L, and appropriate treatments in 3 days were associated with fatal outcomes. A nomogram was developed to predict the survival probability in adult patients with KP infection based on the associated factors identified in the multivariate Cox regression analysis (Figure 4) (dynamic nomogram: https://jihuichen.shinyapps.io/dynnomapp_for_survival/, Supplementary Figure S3). The established nomogram demonstrated strong accuracy and reliability, as indicated by a C-index of 0.86 and a Brier score of 0.109. The ROC curves, calibration plots, and DCA for 7-, 15-, and 30-day further validated the clinical usefulness and consistency of the model (Figure 5). The validation set yielded a C-index of 0.91 and a Brier score of 0.074. The ROC curves, along with the calibration plots, demonstrated good concordance and reliability (Supplementary Figure S4).

**TABLE 4:** Results of univariate and multivariate Cox regression analyses for risk factors associated with 30-day mortality.

Variable^a^	Univariate			Multivariate		
	HR	95% CI	*P* value	HR	95% CI	*P* value
CRKP vs. CSKP	5.30	3.06–9.20	<0.001			
Age, y, ≥65 vs. < 65	2.77	1.60–4.81	<0.001	2.26	1.26–4.06	0.006
CCI, 1-point increments	1.27	1.17–1.37	<0.001	1.10	1.00–1.21	0.046
Renal disease	2.35	1.46–3.78	<0.001			
Respiratory failure	5.70	3.63–8.96	<0.001	3.28	2.05–5.24	<0.001
Heart failure	2.62	1.64–4.18	<0.001			
MODS	7.04	4.14–11.95	<0.001			
SOFA, 1-point increments	1.22	1.16–1.28	<0.001	1.25	1.17–1.34	<0.001
ICU admission	3.72	2.05–6.75	<0.001			
Mechanical ventilation	4.39	2.53–7.62	<0.001			
Central venous catheterization	6.16	2.96–12.8	<0.001			
Urinary catheterization	4.08	1.96–8.49	<0.001			
Gastric catheterization	4.83	2.61–8.94	<0.001			
CRRT	3.68	1.99–6.81	<0.001			
Pneumonia	1.71	1.06–2.76	0.027			
WBC, × 10^9^/L, ≥10 vs. < 10	1.84	1.14–2.96	0.012			
Hemoglobin, g/L, ≤100 vs. > 100	2.96	1.82–4.79	<0.001			
Platelet, × 10^9^/L, ≤100 vs. > 100	3.16	1.96–5.10	<0.001			
CRP, mg/L, ≥100 vs. < 100	1.58	1.01–2.47	0.044			
Albumin, g/L, ≤30 vs. > 30	2.78	1.78–4.35	<0.001	1.54	0.97–2.46	0.067
Appropriate treatments in 3 days	0.22	0.14–0.34	<0.001	0.31	0.19–0.49	<0.001

Notes:

^a^ Variables correlating with *P* < 0.05.

Abbreviations: HR, hazard ratio; CI, confidence interval; CRKP, carbapenem-resistant *Klebsiella pneumoniae*; CRKP, carbapenem-susceptible *Klebsiella pneumoniae*; CCI, charlson comorbidity index; MODS, multiple organ dysfunction syndrome; SOFA, sequential organ failure assessment; ICU, intensive care unit; WBC, white blood count; CRRT, continuous renal replacement therapy; CRP, C-reactive protein.

## Discussion

The present study aimed to identify the risk factors and build nomograms to predict the risk of CRKP infection and mortality based on multivariable logistic and Cox regression analyses in adult patients. The nomogram presents a straightforward and pragmatic clinical system, allowing for the identification of high-risk patients who may benefit from intensive medical intervention. The nomogram used to predict CRKP infection was composed of two variables CCI (a tool to measure comorbidity burden) and prior hospitalization (Xiao et al., 2020) (a well-known risk factor for CRKP infection). Similar to the previous reports (Li et al., 2020; Xiao et al., 2020; Dai et al., 2021; Zuo et al., 2021; Wu et al., 2022), prior exposure to carbapenems and broad-spectrum β-lactams were included in the nomogram and acted as independent risk factors. The use of a central venous catheter was also identified as a risk factor in a previous study, (Li et al., 2020), and particularly, it showed the longest line in the nomogram, implying the greatest impact on the CRKP acquisition risk in our study. ICU admission, SOFA score, mechanical ventilation, urinary catheterization, and gastric catheterization were also associated with CRKP acquisition in the univariate analysis and the previous studies (Liu et al., 2018; Li et al., 2020; Xiao et al., 2020). Because of obvious correlations observed between central venous catheterization and ICU admission (r = 0.70), SOFA score (r = 0.49), mechanical ventilation (r = 0.66), urinary catheterization (r = 0.56), and gastric catheterization (r = 0.66), only central venous catheterization with the highest predictive value was included in the final nomogram. These five variables, included in the final nomogram, were obtained through routine examinations, which offered a prominent advantage. Thus, the nomogram could facilitate the identification of patients at high risk of CRKP infection, thereby informing and guiding decision-making in their clinical management. For high-risk patients, antibiotics such as tigecycline, polymyxin, and ceftazidime-avibactam, may be considered.

In our study, the 30-day mortality of 32.4% for CRKP was much higher than that of the CSKP (6.4%). We performed PSM including variables such as sex, age, baseline disease or comorbidity, CCI, clinical status, invasive procedure, and type of infections to minimize the difference between the two groups and ensure a more accurate assessment of the effects of CRKP. After excluding confounding factors, we found that the 30-day mortality rate was significantly higher in the CRKP group (log-rank *P* = 0.002). Also, in the original dataset, differences in 30-day mortality between the two groups remained significant (adjust-HR, 2.36; 95% CI: 1.09–5.14; *P* = 0.030) after adjusting for the confounding factors in Cox multivariate analysis. The abovementioned results indicated that CRKP infection was directly related to poor prognosis, similar to that reported previously (Kohler et al., 2017; Xu et al., 2017; Yuan et al., 2020).

The risk factors for mortality in patients with KP infection in our analysis were found to be similar to those reported in past publications (Xu et al., 2017; Xu et al., 2018; Agyeman et al., 2020). We accordingly developed a nomogram to predict the prognosis of patients with KP infection and observed that the factors age, CCI, respiratory failure, SOFA score, and albumin were associated with 30-day mortality. Consistent with the previous studies (Xiao et al., 2020; Chen et al., 2021), we found that an appropriate antimicrobial regimen in 3 days (HR, 0.31; 95% CI, 0.19–0.49; *P* < 0.001) significantly improved the prognosis and that the inclusion of the variable significantly improved the predictive power of the model. With the widespread use of mNGS, more KP infections can be diagnosed early, albeit it cannot be confirmed whether the strains are resistant to carbapenems. Therefore, to increase the applicability of the nomogram, we did not include the variable of CRKP, although including CRKP slightly increased the predictive power (C-index, 0.865; 95% CI: 0.832–0.898; *P* = 0.022 by ANOVA analysis). For patients at high-risk, intensive care management and an aggressive antimicrobial regimen should be considered (Qureshi et al., 2012; Chen et al., 2021). In our previous study (Chen et al., 2021), we identified that antibiotics such as ceftazidime-avibactam, fosfomycin, and amikacin have shown promising effectiveness against CRKP infections. However, while tigecycline improved early treatment outcomes, it was associated with a trend toward increased mortality, necessitating cautious use. Combination therapy, particularly in high-risk patients, has demonstrated significant benefits in reducing mortality, underscoring the importance of prompt and appropriate antibiotic regimens to improve prognosis and clinical outcomes.

The present study has several limitations. First, this is a single-center, retrospective, observational study, and hence involves a certain degree of selection bias. Second, the study did not include a molecular analysis to confirm the presence of genes encoding carbapenemase production. Finally, the established nomograms were not validated based on external data, and dynamic nomograms were plotted for more extensive validation.

## Conclusion

We developed nomograms to predict the probability of CRKP infection and mortality in adult patients. CCI ≥3, the use of central venous catheterization, hospitalization during the 3 months before infection, and previous exposure to carbapenems and broad-spectrum β-lactams were found to be independently associated with CRKP infection via logistic analysis. Multivariate Cox regression analysis identified several factors associated with 30-day mortality, including age ≥65 years, higher CCI, the presence of respiratory failure, higher SOFA score, albumin level ≤30 g/L, and appropriate treatments within the first 3 days. The established nomograms indicated good consistency and discrimination that can facilitate clinicians to make better clinical decisions when treating patients with KP infection. Nevertheless, prospective studies of a larger number of patients are needed to validate the predictive value of the models.

## Data Availability

The raw data supporting the conclusions of this article will be made available by the authors, without undue reservation.
