# Supplementary material for: Development of prediction models for carbapenem-resistant Klebsiella pneumoniae acquisition and prognosis in adult patients
Source: Front Pharmacol. 2024 Nov 5;15:1439116. doi: 10.3389/fphar.2024.1439116 (PMC11573532; doi:10.3389/fphar.2024.1439116)
Supplement: Supplementary file 1 [file DataSheet1.PDF]

ORIGINAL RESEARCH

Huijuan Yao and Yu Yang et al

Development of prediction models for carbapenem-resistant Klebsiella pneumoniae acquisition and prognosis in adult patients

Table S1 Characteristics of the study patients with Klebsiella pneumoniae infection in validation cohort.

Characteristic	All patients n=86	CRKP infection n=44 (51%)	CSKP infection n=42 (49%)
**Patient variables**			
Male sex, n (%)	57 (66%)	31 (31%)	26 (64%)
Age, y, median (IQR)	68 (60, 76)	72 (64, 79)	66 (59, 72)
**Baseline disease or comorbidity, n (%)**			
Diabetes mellitus	21 (24%)	7 (16%)	14 (33%)
Cardiovascular disease	42 (49%)	15 (36%)	27 (61%)
Cerebrovascular disease	28 (33%)	8 (19%)	20 (45%)
Renal disease	19 (22%)	4 (9.5%)	15 (34%)
Hematological disease	5 (5.8%)	2 (4.8%)	3 (6.8%)
Digestive diseases	32 (37%)	16 (38%)	16 (36%)
Malignant solid tumor	23 (27%)	13 (31%)	10 (23%)
Prior surgery[a]	53 (62%)	26 (59%)	27 (64%)
Immunosuppressant use	11 (13%)	3 (6.8%)	8 (19%)
ICU admission	49 (57%)	32 (73%)	17 (40%)
**CCI, median (IQR)**	3 (2, 5)	4 (3, 6)	2 (1, 3.75)
CCI≥3, n (%)	51 (59%)	35 (80%)	16 (38%)
**Clinical status, n (%)**			
Respiratory failure	15 (17%)	13 (30%)	2 (4.8%)
Heart failure	15 (17%)	10 (23%)	5 (12%)
MODS	9 (10%)	7 (16%)	2 (4.8%)
SOFA score, median (IQR)	3.0 (1.0, 6.0)	3.5 (2.0, 5.0)	2.0 (0.0, 6.0)
**Invasive procedure, n (%)**			
Mechanical ventilation	44 (51%)	30 (68%)	14 (33%)
Central venous catheterization	49 (57%)	33 (75%)	16 (38%)
Urinary catheterization	65 (76%)	39 (89%)	26 (62%)
Gastric catheterization	46 (53%)	30 (68%)	16 (38%)
CRRT	7 (8.1%)	4 (9.1%)	3 (7.1%)
**Type of infections, n (%)**			
Pneumonia	50 (58%)	29 (66%)	21 (50%)
Intra-abdominal infection	8 (9.3%)	5 (11%)	3 (7.1%)
Urinary tract infection	12 (14%)	5 (11%)	7 (17%)
Gastrointestinal infection	6 (7.0%)	0 (0%)	6 (14%)

Primary bloodstream infection	1 (1.2%)	1 (2.3%)	0 (0%)
Skin and soft-tissue infection	7 (8.1%)	2 (4.5%)	5 (12%)
Catheter-related infection	3 (3.5%)	2 (4.5%)	1 (2.4%)
**Laboratory variables from blood, Mean ± SD**			
WBC, × 10^9/L	10.3 (7.5, 15.2)	10.2 (7.4, 16.0)	10.5 (7.6, 14.7)
ANC, × 10^9/L	8.8 (5.3, 13.8)	8.7 (5.7, 14.5)	8.8 (4.9, 12.7)
Lymphocyte, × 10^9/L	0.94 (0.50, 1.50)	0.88 (0.50, 1.32)	1.05 (0.63, 1.58)
Hemoglobin, g/L	102 (86, 119)	95 (84, 112)	112 (92, 129)
Platelet, × 10^9/L	187 (127, 250)	198 (135, 254)	174 (120, 242)
C-reactive protein, mg/L	49 (17, 119)	64 (29, 131)	39 (10, 96)
Albumin, g/L	33.8 (29.7, 36.4)	31.3 (28.5, 35.1)	34.4 (31.3, 37.8)
Hospital stay before KP infection, days, median (IQR)	9 (4, 15)	12 (6, 17)	6 (3, 11)
**Previous exposure to antibiotics[a], n (%)**			
Carbapenems	66 (77%)	22 (50%)	4 (9.5%)
Broad-spectrum β-lactams[b]	26 (30%)	34 (77%)	13 (31%)
First and second-generation cephalosporins	47 (55%)	0 (0%)	2 (4.8%)
Fluoroquinolones	2 (2.3%)	15 (34%)	6 (14%)
Aminoglycosides	21 (24%)	8 (18%)	0 (0%)
Tigecyclines	8 (9.3%)	6 (14%)	0 (0%)
Glycopeptides	6 (7.0%)	0 (0%)	7 (17%)
Fosfomycins	7 (8.1%)	5 (11%)	1 (2.4%)
Tetracyclines	6 (7.0%)	4 (9.1%)	0 (0%)
Prior hospitalization[c], n (%)	27 (31%)	19 (43%)	8 (19%)
30-day mortality, n (%)	13 (15%)	11 (25%)	2 (4.8%)
Total hospitalisation cost[d], USD, Mean ± SD	27,786 (13,040, 130,740)	38,050 (16,148, 199,093)	23,588 (10,696, 50,008)

Notes: [a] During the 30 days before *Klebsiella pneumoniae* infection; [b] Broad-spectrum β-lactams including third- and fourth-generation cephalosporins, beta-lactam: beta-lactamase inhibitor combinations; [c] During the 3 months before *Klebsiella pneumoniae* infection;[d] USD1 = CNY6.5 in year 2021

Abbreviations: CRKP, carbapenem-resistant Klebsiella pneumoniae; CRKP, carbapenem-susceptible Klebsiella pneumoniae; IQR, interquartile Range; MODS, multiple organ dysfunction syndrome; ICU, intensive care unit; CCI, Charlson comorbidity index; SOFA, Sequential Organ Failure Assessment; CNS, central nervous system; CRRT, continuous renal replacement therapy; WBC, white blood count; ANC, absolute neutrophil count

Table S2. Characteristics of Patients with *Klebsiella pneumoniae* Infections after Propensity Score Matching

Characteristic	All patients n = 129	CRKP infection n = 53	CSKP infection n= 76	*P* value
**Patient variables**				
Male sex, n (%)	80 (62.0)	31 (58.5)	49 (64.5)	0.491
Age, y, median (IQR)	68 (59, 77)	71 (59, 82)	67 (58.5, 73)	0.367
**Baseline disease or comorbidity, n (%)**				
Diabetes mellitus	47 (36.4)	16 (30.2)	31 (40.8)	0.220
Cardiovascular disease	57 (44.2)	21 (39.6)	36 (47.4)	0.384
Cerebrovascular disease	41 (31.8)	19 (35.8)	22 (28.9)	0.408
Hematological disease	27 (20.9)	12 (22.6)	15 (19.7)	0.690
Digestive diseases	12 (9.3)	7 (13.2)	5 (6.6)	0.230
Malignant solid tumor	37 (28.7)	15 (28.3)	22 (28.9)	0.936
Prior surgery[a]	29 (22.5)	7 (13.2)	22 (28.9)	0.053
Immunosuppressant use	77 (59.7)	33 (62.3)	44 (57.9)	0.619
ICU admission	83 (64.3)	37 (69.8)	46 (60.5)	0.280
**CCI, median (IQR)**	3 (2, 4)	3 (2, 4)	3 (2, 4)	0.322
**Clinical status, n (%)**				
Respiratory failure	19 (14.7)	11 (20.8)	8 (10.5)	0.113
MODS	12 (9.3)	6 (11.3)	6 (7.9)	0.512
SOFA score, median (IQR)	4 (1, 7)	4 (1, 7)	3 (0, 6.25)	0.445
**Invasive procedure and/or devices, n (%)**				
Mechanical ventilation	63 (48.8)	27 (50.9)	36 (47.4)	0.689
Central venous catheterization	91 (70.5)	40 (75.5)	51 (67.1)	0.307
Urinary catheterization	96 (74.4)	39 (73.6)	57 (75.0)	0.856
Gastric catheterization	73 (56.6)	32 (60.4)	41 (53.9)	0.469
CRRT	6 (4.7)	4 (7.5)	2 (2.6)	0.228
**Type of infections, n (%)**				
Catheter-related infection	4 (3.1)	3 (5.7)	1 (1.3)	0.305
Pneumonia	67 (51.9)	24 (45.3)	43 (56.6)	0.208
Intra-abdominal infection	7 (5.4)	4 (7.5)	3 (3.9)	0.445
Urinary tract infection	26 (20.2)	13 (24.5)	13 (17.1)	0.303
Gastrointestinal infection	12 (9.3)	3 (5.7)	9 (11.8)	0.357
Primary bloodstream infection	11 (8.5)	5 (9.4)	6 (7.9)	0.759
Skin and soft-tissue infection	5 (3.9)	3 (5.7)	2 (2.6)	0.401
CNS infection	1 (0.8)	0 (0)	1 (1.3)	1

[a]During the 30 days preceding *Klebsiella pneumoniae* infection

Abbreviations: CRKP, carbapenem-resistant *Klebsiella pneumoniae*; CSKP, carbapenem-susceptible *Klebsiella pneumoniae*; IQR, interquartile Range; ICU, intensive care unit; CCI, Charlson comorbidity index; MODS, multiple organ dysfunction syndrome; SOFA,

Sequential Organ Failure Assessment; CRRT, continuous renal replacement therapy; CNS, central nervous system

Table S3. Characteristics of Patients with *Klebsiella pneumoniae* Infections

Characteristic	30-day non-survivors n = 84 (18.0%)	30-day survivors n = 383 (82.0%)	*P* value
**CRKP, n (%)**	68 (81.0)	142 (37.1)	<0.001
**Patient variables**			
Male sex, n (%)	57 (67.9)	264 (68.9)	0.848
Age, y, median (IQR)	75 (65.75, 84)	66 (57, 74)	<0.001
**Baseline disease or comorbidity, n (%)**			
Diabetes mellitus	28 (33.3)	125 (32.6)	0.902
Cardiovascular disease	47 (56.0)	161 (42.0)	0.021
Cerebrovascular disease	36 (42.9)	136 (35.5)	0.207
Renal disease	28 (33.3)	58 (15.1)	<0.001
Hematological disease	5 (6.0)	18 (4.7)	0.632
Digestive diseases	25 (29.8)	128 (33.4)	0.518
Malignant solid tumor	19 (22.6)	91 (23.8)	0.823
Prior surgery[a]	51 (60.7)	217 (56.7)	0.496
Immunosuppressant use	8 (9.5)	55 (14.4)	0.244
ICU admission	68 (81.0)	208 (54.3)	<0.001
**CCI, median (IQR)**	4.5 (3, 6)	3 (2, 4)	<0.001
**Clinical status, n (%)**			
Respiratory failure	32 (38.1)	38 (9.9)	<0.001
Heart failure	30 (35.7)	57 (14.9)	<0.001
MODS	21 (25.0)	7 (1.8)	<0.001
SOFA score, median (IQR)	5 (3, 9)	2 (0, 4)	<0.001
**Invasive procedure and/or devices, n (%)**			
Mechanical ventilation	65 (77.4)	164 (42.8)	<0.001
Central venous catheterization	73 (86.9)	212 (55.4)	<0.001
Urinary catheterization	75 (89.3)	250 (65.3)	<0.001
Gastric catheterization	68 (81.0)	191 (49.9)	<0.001
CRRT	12 (14.3)	15 (3.9)	0.001
**Type of infections, n (%)**			
Catheter-related infection	1 (1.2)	8 (2.1)	1
Pneumonia	57 (67.9)	205 (53.5)	0.018
Intra-abdominal infection	6 (7.1)	26 (6.8)	0.907
Urinary tract infection	12 (14.3)	77 (20.1)	0.221
Gastrointestinal infection	1 (1.2)	36 (9.4)	0.007
Primary bloodstream infection	7 (8.3)	21 (5.5)	0.323
Skin and soft-tissue infection	1 (1.2)	14 (3.7)	0.490
CNS infection	0 (0)	2 (0.5)	1
**Laboratory variables from blood,**			

**Mean ± SD**			
WBC, $\times 10^9$/L	12.83±6.44	10.79±5.15	0.002
ANC, $\times 10^9$/L	18.97±23.52	9.34±7.78	<0.001
Lymphocyte, $\times 10^9$/L	7.13±6.64	4.43±6.11	0.001
Hemoglobin, g/L	94.48±20.3	106.32±23.12	<0.001
Platelet, $\times 10^9$/L	170.93±115.58	228.11±122.27	<0.001
CRP, mg/L	97.35±64.08	78.31±61.54	0.012
Albumin, g/L	31.73±6.86	33.56±5.2	0.007
Hospital stay before KP infection, days, median (IQR)	11 (5, 20)	8 (4, 16)	0.037
Prior hospitalization[b], n (%)	43 (51.2)	164 (42.8)	0.163
Appropriate treatments in 3 days, n (%)	41 (48.8)	330 (86.2)	<0.001
Antimicrobial regimens, n (%)			
Carbapenems	43 (51.2)	130 (33.9)	0.003
cefoperazone-sulbactam or piperacillin-tazobactam	26 (31.0)	131 (34.2)	0.568
Broad spectrum β-lactams	12 (14.3)	97 (25.3)	0.033
Ceftazidime-avibactam	1 (1.2)	12 (3.1)	0.479
Tigecycline	24 (28.6)	51 (13.3)	0.001
Polymyxin B	3 (3.6)	12 (3.1)	0.837
Aminoglycosides	6 (7.1)	41 (10.7)	0.329
Fosfomycin	9 (10.7)	51 (13.3)	0.520
Fluoroquinolones	6 (7.1)	61 (15.9)	0.043
Total hospitalisation cost[c], USD, Mean ± SD	32024±31233	25251±40036	0.178
Total hospital days, median (IQR)	20 (12, 35)	22 (12, 38)	0.915

[a]During the 30 days preceding *Klebsiella pneumoniae* infection

[b]During the 3 months preceding *Klebsiella pneumoniae* infection

[c]USD1 = CNY6.5 in year 2021

Abbreviations: CRKP, carbapenem-resistant *Klebsiella pneumoniae*; IQR, interquartile Range; ICU, intensive care unit; CCI, Charlson comorbidity index; MODS, multiple organ dysfunction syndrome; SOFA, Sequential Organ Failure Assessment; CRRT, continuous renal replacement therapy; CNS, central nervous system; WBC, white blood count; ANC, absolute neutrophil count; CRP, c-reactive protein

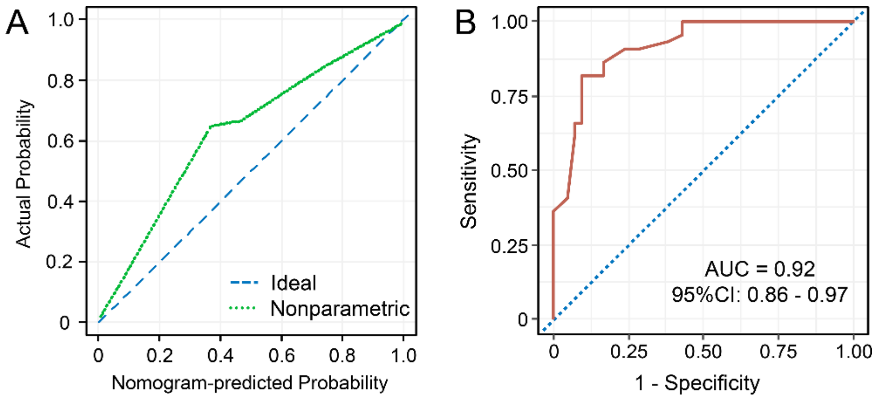

Figure S1. Calibration curves (A) and the receiver operating characteristics (ROC) curves (B) for the nomogram to predict the probability of carbapenem-resistant Klebsiella pneumoniae in validation cohort. Abbreviations: AUC, the area under the curve; CI, confidence interval.

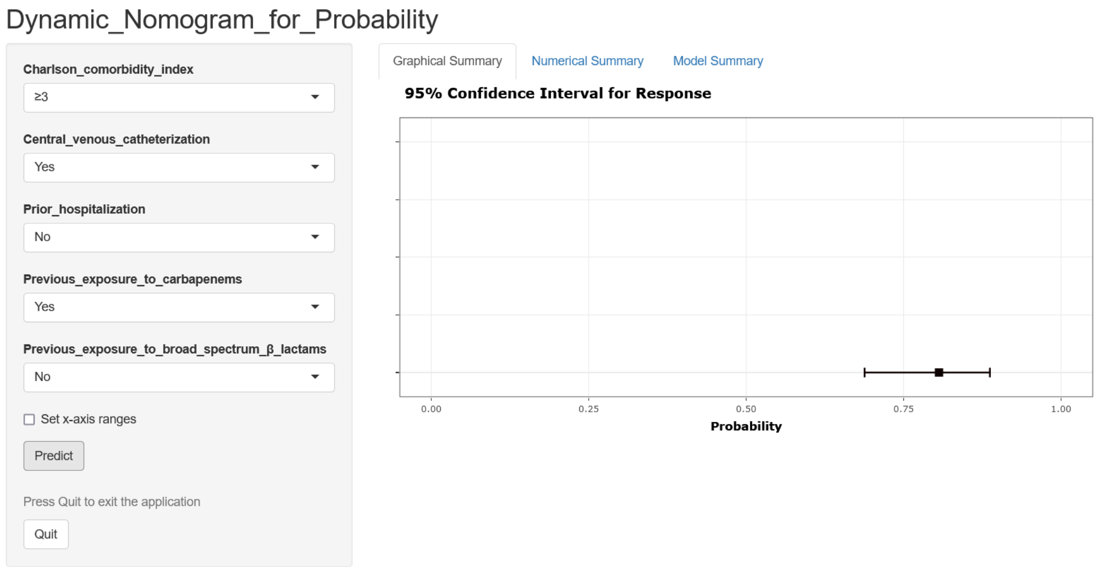

Figure S2. The dynamic nomogram to predict the probability of CRKP infection.

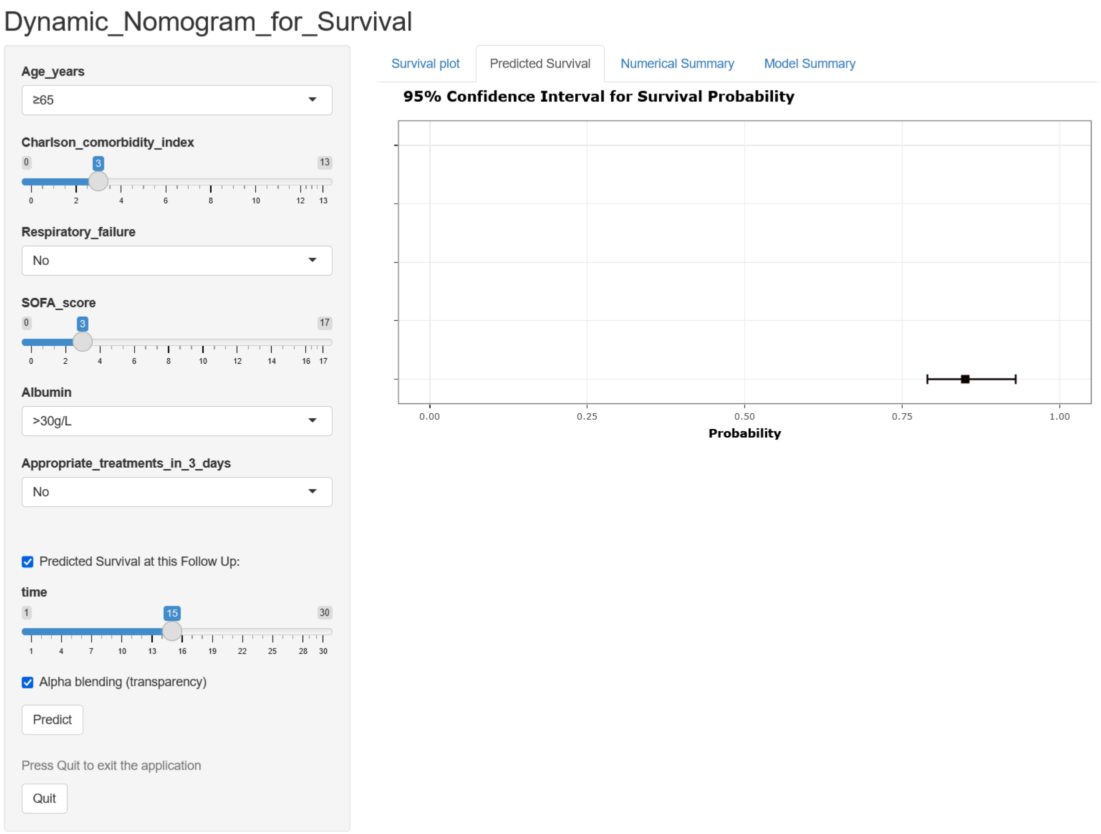

Figure S3. The dynamic nomogram to predict the survival probability of KP infection patients.

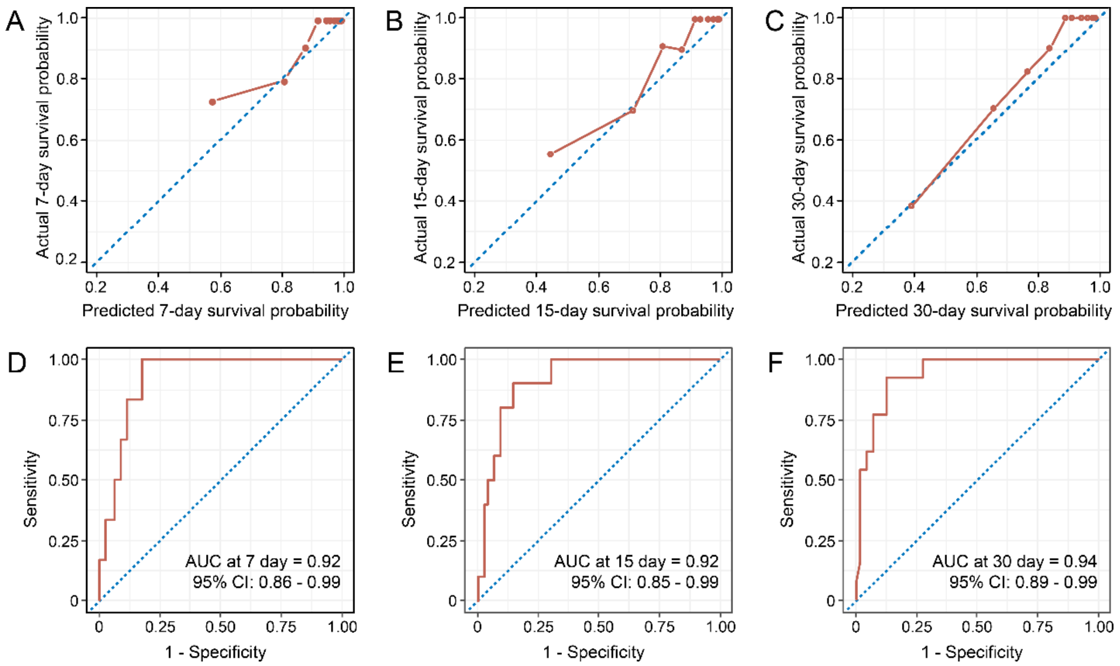

Figure S4. Calibration curves (A) and the time-dependent receiver operating characteristics (ROC) curves (B) for the nomogram predicting 7-, 15-, and 30-day survival probability in validation cohort. Abbreviations: AUC, the area under the curve; CI, confidence interval.
